# Radiomics for Everyone: A New Tool Simplifies Creating Parametric Maps for the Visualization and Quantification of Radiomics Features

**DOI:** 10.3390/tomography7030041

**Published:** 2021-09-17

**Authors:** Damon Kim, Laura J. Jensen, Thomas Elgeti, Ingo G. Steffen, Bernd Hamm, Sebastian N. Nagel

**Affiliations:** 1 Charité—Universitätsmedizin Berlin, Corporate Member of Freie Universität Berlin and Humboldt-Universität zu Berlin, Klinik für Radiologie, Hindenburgdamm 30, 12203 Berlin, Germany; laura-jacqueline.jensen@charite.de (L.J.J.); thomas.elgeti@charite.de (T.E.); ingo.steffen@charite.de (I.G.S.); Bernd.hamm@charite.de (B.H.); sebastian.nagel@charite.de (S.N.N.); 2 Charité—Universitätsmedizin Berlin, Corporate Member of Freie Universität Berlin and Humboldt-Universität zu Berlin, Klinik für Radiologie, Arbeitsbereich Kinderradiologie, Augustenburger Platz 1, 13353 Berlin, Germany

**Keywords:** image enhancement, diagnostic techniques and procedures, image processing, computer-assisted

## Abstract

Aim was to develop a user-friendly method for creating parametric maps that would provide a comprehensible visualization and allow immediate quantification of radiomics features. For this, a self-explanatory graphical user interface was designed, and for the proof of concept, maps were created for CT and MR images and features were compared to those from conventional extractions. Especially first-order features were concordant between maps and conventional extractions, some even across all examples. Potential clinical applications were tested on CT and MR images for the differentiation of pulmonary lesions. In these sample applications, maps of Skewness enhanced the differentiation of non-malignant lesions and non-small lung carcinoma manifestations on CT images and maps of Variance enhanced the differentiation of pulmonary lymphoma manifestations and fungal infiltrates on MR images. This new and simple method for creating parametric maps makes radiomics features visually perceivable, allows direct feature quantification by placing a region of interest, can improve the assessment of radiological images and, furthermore, can increase the use of radiomics in clinical routine.

## 1. Introduction

Radiomics are an emerging means in image analysis [1,2,3,4] that allow quantitative image assessment beyond morphologic and macroscopic characteristics [5]. For this, statistics of the grey level composition in a region of interest (ROI) are calculated, resulting in many different quantitative texture features that can be statistically analyzed and linked to an outcome [5]. Numerous studies have shown the potential of radiomics in the differentiation of various pathological entities [6,7,8,9]. Thus, the spectrum of possible applications is huge. A very specific application, for example, is the differentiation of pulmonary lymphoma manifestations and non-lymphoma infiltrates in suspected fungal pneumonia in hematooncologic patients [10]. In this collective, the first-order feature Variance has shown to be a useful parameter [11]. However, regardless of the application, all approaches so far typically require image segmentation before feature extraction, and often, basic programming skills can be of help [12]. Finally, the process usually results in exclusively abstract numerical values.

A recent article by Pinto dos Santos et al. discussed the translational gap of radiomics into clinical routine and saw the lack of reliable and reproducible results from high-evidence studies as one of the main reasons [13]. We believe that the usability of the method should also be simplified. Beyond that, a graphical representation of features in parametric maps and fusion images would visualize features in a comprehensible way, which could help to easier understand the information they convey and, thus, to draw immediate conclusions.

The aim of this project, therefore, was to develop a straightforward, user-friendly method for creating parametric maps. For the proof of concept, feature values retrieved from the maps were compared to those from conventional extractions. Potential clinical applications were tested on CT and MR images for the differentiation of pulmonary lesions.

## 2. Materials and Methods

### 2.1. Concept of the Parametric Map Creation Tool

Creating the parametric maps should be as simple as possible. The basic idea was that the user chooses a folder containing an examination in DICOM-format, adjusts the extraction settings, clicks “start” and receives the desired parametric map(s) in DICOM-format. We chose Python as the programming language, because it is platform-independent, and since the feature extraction should be done with PyRadiomics [14], no cross-language interfaces would be required.

**Figure 1:**
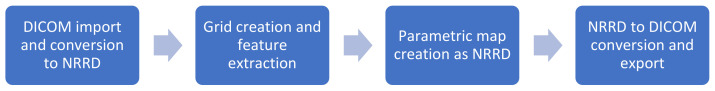
Summarized workflow of the program up to the final parametric map.

**Figure 2:**
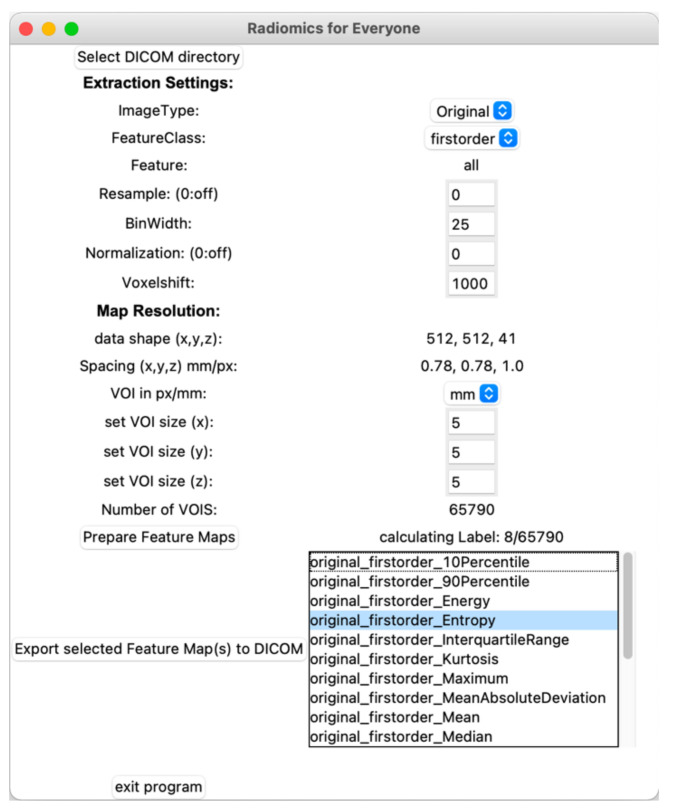
Graphical user interface. After importing the original images, the user can adjust settings for the feature extraction and define the resolution of the parametric map. Once the features are calculated, parametric maps can be exported in the DICOM format by choosing the feature from a list.

Figure 1 summarizes the workflow of the program up to the final parametric map. After import, the examination image data are converted to the NRRD-format (“nearly raw raster data”) using simpleITK [15]. A second NRRD-file of the same dimensions is created to contain a grid of volumes of interest (VOI), i.e., a grid that divides the image into small blocks. These VOIs, in turn, are produced by three cascaded loops, each for each spatial dimension (x-, y-, *z*-axis). The VOI size, and thus the resolution of the resulting parametric map, can be defined by the user either pixel- or voxel-wise. Each VOI is assigned a unique, incremental ID. Figure 2 shows the graphical user interface with settings for the feature extraction and the resolution of the parametric map. After the grid is generated, the image-NRRD and the grid-NRRD are parsed to PyRadiomics for the actual feature calculation for each VOI. The results are stored in a CSV-file with each row representing a single VOI and each column displaying a different feature value. In the next step, the parametric map is created by filling the grid-NRRD with the data from the CSV-file by matching the VOI-ID. The resulting feature map is then reconverted to the DICOM file format by again using simpleITK and is now readable by standard image viewers. If supported by the viewer, parametric maps can also be used as an overlay to anatomic images in fusion images.

### 2.2. Settings for PyRadiomics

Configuring PyRadiomics for the feature extraction allows adjustment of a vast number of parameters. As the intention was to design a tool for the easy creation of parametric maps, we provide options to adjust basic settings. However, a future version of the tool could include a section for advanced settings.

### 2.3. Proof of Concept Examples

To demonstrate the concept, sample maps for all features available in PyRadiomics were created. For CT images, these samples included a ROI in segments VII/VIII in an otherwise unremarkable liver, where parametric maps were created with two different resolutions. Another CT was considered of a bronchial carcinoma. For MR images, a glioblastoma and a hepatocellular carcinoma (HCC) were included. Details of the feature extraction and image acquisition are given in electronic Tables S1–S4. The same settings and ROIs for both the conventional extraction and the parametric maps were used for corresponding images. Values obtained from the maps and the conventional extraction were then compared feature by feature in tables, and features with a deviation between −20% to +20% from the conventional extraction were highlighted and considered concordant.

### 2.4. Evaluation of Clinical Application

Two potential clinical applications were investigated. For CT images, the differentiation of non-malignant lesions and non-small cell lung carcinoma (NSCLC) manifestations was evaluated on 12 patients from the Lung Image Database Consortium (LIDC) and Image Database Resource Initiative (IDRI) dataset [16] (Patient IDs non-malignant: 0162, 0183, 0221, 0257, 0277, 0510; NSCLC: 0163, 0203, 0242, 0267, 0454, 0470).

For MR images, the differentiation of pulmonary lymphoma manifestations and non-lymphoma infiltrates in suspected fungal pneumonia in T1-weighted thoracic MR images was evaluated in 12 hematooncologic patients (six lymphoma manifestations and six non-lymphoma infiltrates in suspected fungal pneumonia; characteristics shown in electronic Table S5). The images were acquired on a clinical scanner (Magnetom Skyra, Siemens Healthineers, Erlangen, Germany; Volumetric interpolated breath-hold examination [VIBE], slice 3 mm, TR 5.4 ms, TE 2.0 ms, Flip 9°, matrix 320 × 195, individual field of view). Standard of reference was histopathologic workup or clinical diagnosis including criteria according to the European Organization for Research and Treatment of Cancer/Invasive Fungal Infections Cooperative Group and the National Institute of Allergy and Infectious Diseases Mycoses Study Group (EORTC/MSG) [17]. The patients were part of a collective reported before [18].

To identify relevant features, first a conventional segmentation and feature extraction was performed. The results were used to define cutoff values in a ROC analysis using Youden’s index [19]. For simplicity, only the best-performing feature was considered.

During the actual reading, the lesions were first rated based on morphologic criteria (CT: non-malignant, NSCLC, unclear; MR: non-lymphoma, lymphoma, unclear). Afterwards, the mean value of the previously determined feature was retrieved from the corresponding map with a manually drawn ROI and the lesion was rated only by considering the cutoff value. Time to diagnosis during the reading was assessed. The primary reading was done by an experienced, board-certified radiologist (S.N.N., with 10 years of experience) and repeated after 6 weeks to assess intrarater reliability. To test for interrater reliability, two additional readings were done by two experienced radiology residents (D.K., with 5 years of experience, and L.J.J., with 4 years of experience).

To assess the inter- and intrarater reliability of the qualitative ratings based on morphologic criteria, Fleiss’ and Cohen’s Kappa were calculated and rated according to Landis and Koch (0.00–0.20 slight, 0.21–0.40 fair, 0.41–0.60 moderate, 0.61–0.80 substantial, 0.81–1.00 almost perfect) [20].

To assess interrater agreement of the quantitative feature values, intraclass correlation coefficient (ICC) estimates and their 95% confidence intervals were calculated based on a mean-rating (k = 3), absolute-agreement, two-way random-effects model. For assessment of intrarater agreement, ICC estimates and their 95% confidence intervals were calculated based on a mean-rating (k = 2), absolute-agreement, two-way mixed-effects model. Intra- and interrater reliability was rated according to Koo et al. (ICC < 0.5, poor; 0.5–0.75, moderate; 0.75–0.9, good; >0.9, excellent) [21].

## 3. Results

A tool with a graphical user interface was designed and works as desired: the user can select a DICOM-folder, adjust settings, and receive a parametric map in DICOM format.

### 3.1. Proof of Concept Examples

**Figure 3:**
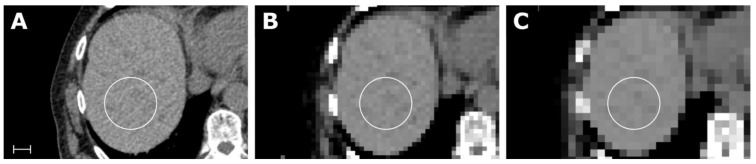
Samples of an abdominal CT (**A**) with corresponding maps for the feature Root Mean Squared (RMS), with a resolution of 5 × 5 × 5 mm in (**B**) and 10 × 10 × 10 px in (**C**). Scale indicates 2 cm. The value for RMS in the liver parenchyma from the conventional extraction and for both maps was 1060. Details of the scan and feature extraction as well as a full list of all values is available in electronic Table S1.

All parametric maps were successfully created. The first-order feature Root Mean Squared is shown in Figure 3 for the ROI in liver segments VII/VIII. To see a full list of all values, please review electronic Table S5. Considering the feature class, first-order and GLCM, and considering the map resolution, 10 × 10 × 10 px (i.e., the lower resolution) revealed more concordant feature values in relation to the conventional extraction.

**Figure 4:**
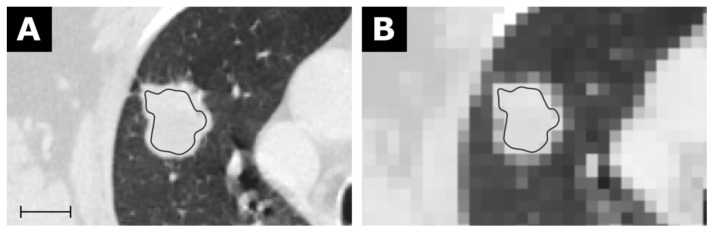
CT of a bronchial carcinoma (**A**) with a corresponding map for the feature Mean with a resolution of 5 × 5 × 5 mm in (**B**). Scale indicates 2 cm. The value for Mean from the conventional extraction was 24.9 and 22.2 for the map. Details are available in electronic Table S2.

**Figure 5:**
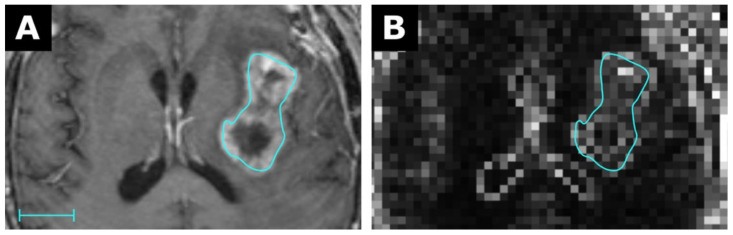
MRI of a glioblastoma (**A**) with a corresponding map for the feature Robust Mean Absolute Deviation (RMAD) with a resolution of 3 × 3 × 3 mm in (**B**). Scale indicates 2 cm. The value for RMAD from the conventional extraction was 42.3 and 18.0 for the map. Details are available in electronic Table S3.

**Figure 6:**
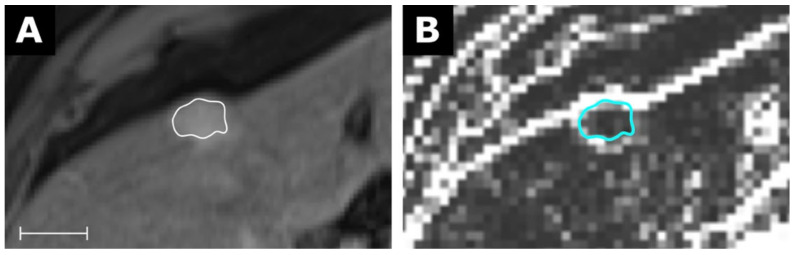
MRI of a hepatocellular carcinoma (**A**) with a corresponding map for the feature Variance with a resolution of 3 × 3 × 3 mm in (**B**). Scale indicates 2 cm. The value for Variance from the conventional extraction was 882 and 108 for the map. Details are available in electronic Table S4.

As further examples, the first-order feature Mean is exemplary shown in Figure 4 for the CT of a bronchial carcinoma, the first-order feature Robust Mean Absolute Deviation for the MRI of a glioblastoma in Figure 5, and the first-order feature Variance for the MRI of an HCC in Figure 6.

For the CT of a bronchial carcinoma, the feature class first-order and GLCM again revealed the highest number of concordant feature values, although the total number of features with a deviation between −20% and +20% was lower than for the example considering liver segments VII/VIII. For the MR images, concordant feature values tended to include more higher-order features. Considering all CT and MR images, eight features still pertained concordant values. The stability of all features is summarized in electronic Table S6.

### 3.2. Clinical Application

For the CT images, the feature Skewness was identified, and the cutoff value was set at −0.11 (area under the curve (AUC) 0.83, *p* < 0.05; positive test result: NSCLC), where NSCLC manifestations showed lower and benign lesions higher values.

For the MR images, the feature Variance was identified and the cutoff value was set at 1363 (AUC 0.89, *p* < 0.001; positive test result: lymphoma), where lymphoma manifestations showed lower and non-lymphoma infiltrates showed higher values.

For the CT images, the diagnosis based on morphological criteria was correct in six cases, wrong in three cases and remained unclear in three cases. Using the cutoff value on parametric maps led to 10 correct and two false diagnoses (two benign lesions showed lower values of Skewness, i.e., they were falsely positive classified as malignant). Time to diagnosis using the maps was only dependent on the time drawing the ROI (mean: 5 s), while the morphologic interpretation took longer on average (mean: 17 s) and did not always lead to a decision.

**Figure 7:**
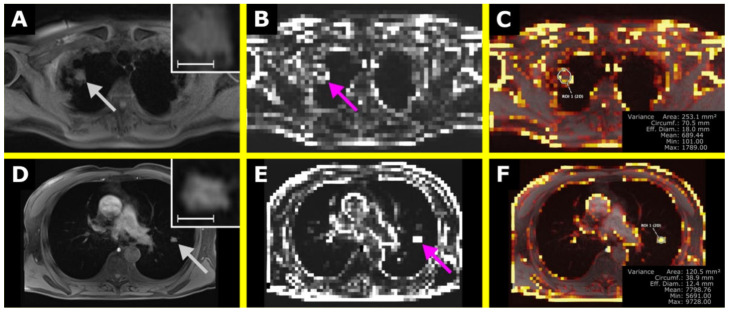
The upper row shows a pulmonary lymphoma manifestation in the right upper lobe with the T1-weighted (T1w) image in (**A**) and the corresponding map of Variance in (**B**). In (**C**), an overlay was created, also showing a ROI to retrieve Variance within the lesion. The lower row shows a non-lymphoma infiltrate in suspected fungal pneumonia in the left upper lobe with the T1w image in (**D**), the corresponding map of Variance in (**C**), and the overlay in (**F**). In (**A**,**D**), the lesions are shown in a magnified section in the upper right corner. The scale indicates 1 cm. (**E**) is the corresponding map of Variance of (**D**). The VISAGE Viewer was used to create the overlay map and to perform the measurements (Visage Imaging Client 7.1.15; Visage Imaging GmbH, Berlin, Germany).

For the MR images, the diagnosis based on morphological criteria was correct in eight cases, wrong in two cases, and remained unclear in two cases. Using the cutoff value on parametric maps led to 10 correct and two false diagnoses (one non-lymphoma infiltrate showed lower values of Variance, i.e., was falsely positive classified as lymphoma, and one pulmonary lymphoma manifestation showed higher values, i.e., was falsely negative classified as non-lymphoma infiltrate). Time to diagnosis using the maps was only dependent on the time drawing the ROI (mean: 5 s), while the morphologic interpretation took longer on average (mean: 14 s) and did not always lead to a decision. Examples are shown in Figure 7.

For the CT images, intrarater reliability was substantial (kappa 0.67) and interrater variability was moderate (kappa 0.59) considering morphological criteria only. Considering the extracted values of Skewness, both intra- and interrater reliability were excellent (ICC 0.99 each; *p* < 0.001).

For the MR images, intrarater and interrater reliability was substantial (kappa 0.67 and 0.79, respectively) considering morphological criteria only. Considering the extracted values of Variance, both intra- and interrater reliability were excellent (ICC 0.98 and 0.99, respectively; *p* < 0.001).

## 4. Discussion

A simplified and user-friendly method to create parametric maps for the visualization and quantification of radiomics features was developed. This approach is suitable for everyday use and does not require programming skills. If overlay maps are used in fusion images, they allow the simultaneous assessment of texture features and morphological criteria, provide a more comprehensive perception, and allow for an immediate quantification. For example, as shown above, fusion images can improve the differentiation of non-malignant pulmonary lesions and NSCLC manifestations on CT images or of pulmonary lymphoma manifestations and non-lymphoma infiltrates in suspected fungal pneumomia on MR images, while also shortening the time to diagnosis.

We consider a visual presentation and immediate assessment of radiomics features important for their inclusion in the diagnostic workflow. If radiologists had parametric maps for radiomics features directly at hand when reporting and if standard values or cutoffs to different entities or conditions were known, simply placing a ROI could be very helpful in making a diagnosis. Until now, a radiomics analysis usually requires several steps from image segmentation to feature extraction and ultimately provides only numeric data without a visual representation.

### 4.1. Parameter Selection

The concept of radiomics consists of a large number of features, which also means that simply applying all of them will not be expedient. Rather, the selection must be made based on the question, e.g., the differentiation of pulmonary lymphoma manifestations and non-lymphoma infiltrates was chosen as an example, because a recent study required only the feature Variance [11]. Our approach would also allow to combine features in a radiomic signature [22], which in turn could be visualized in another, specific parametric map: since the numerical values for each VOI are stored, calculations could be made and results represented in an additional map.

A general downside of radiomics studies is outlined in a literature review by Chetan and Gleeson, in which “the same radiomic feature was rarely identified as being predictive of treatment response in NSCLC by more than one study. This is partly explained by the extensive heterogeneity between individual studies” [23] and is furthermore in line with concerns raised by Pinto dos Santos et al., as outlined in the introduction [13]. Thus, if parametric maps were to be used in clinical routine, further studies would be needed to identify reproducible features suitable for specific settings.

Our results, however, revealed concordant feature values both within the imaging modalities, but also even across all CT and MR images. Interestingly, four GLCM features (ID, IDM, IDMN, Inverse Variance) and one GLSZM feature (Small Area Emphasis) were among them aside from three first-order features (Mean, Median, Root Mean Squared). Against this background, these features may be particularly suitable for parametric maps. Yet, any change in the combination of scanners, settings, images, etc. can lead to different results.

### 4.2. Parametric Map Resolution/VOI Size

The VOI size can be arbitrarily chosen by the user resulting in varying resolutions of the map. While a higher resolution can be desirable, this is associated with higher computing time. A lower resolution, on the other hand, might fail to reasonably assess smaller lesions. One future approach in this regard might be real-time adjustment of the resolution, e.g., with lower resolutions used for the detection of lesions and higher resolutions for their evaluation. In addition to this, the VOI size might directly affect specific parameters known to be confounded by volume (e.g., Energy) [24].

Of course, the extracted features do not represent a single structure or lesion, because the VOIs are solely defined by the grid. Hence, most VOIs will comprise parts of different structures while not including the whole lesion. It is known that features already vary when the edge or core region of a lesion is considered [25]. Against this background, values represented within the parametric maps, even when attributable to a single lesion, are unlikely to be identical to those from a dedicated ROI of the same lesion. Nevertheless, especially values for first-order features from the parametric maps and from the conventional extractions were concordant. Beyond that, the mere correlation of anatomic information with a visual representation of radiomics features could already be considered a key advantage of this approach.

The use of a cutoff values led to two incorrect diagnoses in each of our small studies of potential clinical applications. Reviewing those cases showed that one of the misdiagnosed lesions on the CT images was subsolid, what may have relevantly influenced Skewness, and the other, defined as “unknown”, was very large, and thus, may not even be falsely classified by the cutoff. On the MR images, the misdiagnosed lymphoma manifestation was located centrally and adjacent to many different structures (i.e., blood vessels, bronchi, fat, bone) and also showed slight motion artifacts, which likely increased the level of Variance. On the other hand, the misdiagnosed non-lymphoma infiltrate was located peripherally, and the patient already received treatment for over a week, which made the lesion already appear very homogenous to the naked eye. Nevertheless, we would like to point out that we still consider the results with AUCs of 0.83 and 0.89 very promising, as they were achieved in this very first approach without any prior experience and without any finetuning of the settings. Values retrieved from the parametric maps furthermore showed excellent intra- and interrater for both CT and MR images as opposed to only substantial intra- and moderate interrater reliability for CT and substantial intra- and good interrater reliability for MR images for assessment based on morphologic criteria.

Although no direct comparison to the results of other studies is possible, a study by Baeßler et al. also showed an improvement in diagnosis of chronic vs. acute heart failure-like myocarditis, by applying texture analysis to myocardial T1 and T2 maps vs. applying the maps alone (AUC up to 0.85 vs. 0.51) [26].

### 4.3. Computing Time

Since the computing time depends on many parameters, it is not possible to make a general statement. As a rule of thumb, the calculation time can be estimated by multiplying the total number of voxels in the grid with the time required for a conventional extraction from a single region of interest. However, since the number of voxels, e.g., in a segmented tumor, is likely larger than that in a grid-voxel, one can expect the true calculation time to be slightly shorter.

Basically, of course, the higher the resolution of the grid and underlying image, the higher the computing time. For example, the maps for MR images were calculated faster than those for CT images, which can be explained by their different intrinsic resolutions, especially slice thickness. The complexity of the features is also different, which again results in different computing requirements. In order to reduce the computing time, only relevant blocks of slices from the original images were used in our examples.

### 4.4. Limitations

Without a doubt, the process of creating the parametric maps can be further simplified for the clinical application, e.g., by pre-processing images in the background to provide maps once a study is opened for reporting. This way, no third-party software would be necessary for the radiologist at all.

In addition, involving young residents in the evaluation of a new method may relevantly influence the results of inter- and intrarater reliability. In this study, however, both residents were advanced, so no overall influence from different experience levels is to be expected.

We would also like to underline that while the program used here was written with the greatest possible care and tested for different settings, we cannot rule out bugs. Rather, the program must be considered an early alpha version.

Furthermore, various fields of application, various radiomics features, and various ways to display the parametric maps exist; thus, any change in the setup can affect the output of this method. In consequence, standards would have to be evaluated and defined for specific diagnoses and differential diagnoses, specific imaging modalities, and technical and vendor-specific parameters as well as the reconstruction algorithm.

## 5. Conclusions

We developed a new tool that provides a simple method for creating parametric maps that makes radiomics features visually perceivable and immediately quantifiable. This approach can improve the assessment of radiological images and, furthermore, increase the use of radiomics in clinical routine.

## Data Availability

Part of a publicly available dataset was analyzed in this study. This data can be found in the Lung Image Database Consortium (LIDC) and Image Database Resource Initiative (IDRI) dataset [16].

## Supplementary Materials

The following are available online at https://www.mdpi.com/article/10.3390/tomography7030041/s1, Table S1: CT of unremarkable liver, Table S2: CT of bronchial carcinoma, Table S3: MR of glioblastoma, Table S4: MR of hepatocellular carcinoma, Table S5: Characteristics of the patients considered to test a clinical application for MR images (pulmonary lymphoma manifestations vs. non-lymphoma infiltrates in suspected fungal pneumonia), Table S6: list of concordant features across all examples, Program: Radiomics for Everyone.
